# Inhibitory Effects of *Viscum coloratum* Extract on IgE/Antigen-Activated Mast Cells and Mast Cell-Derived Inflammatory Mediator-Activated Chondrocytes

**DOI:** 10.3390/molecules22010037

**Published:** 2016-12-28

**Authors:** Jae-Myung Yoo, Ju-Hye Yang, Young Soo Kim, Hye Jin Yang, Won-Kyung Cho, Jin Yeul Ma

**Affiliations:** Korean Medicine (KM)-Application Center, Korea Institute of Oriental Medicine (KIOM), 70 Cheomdan-ro, Dong-gu, Daegu 41062, Korea; jmyoo@kiom.re.kr (J.-M.Y.); jjuhye@kiom.re.kr (J.-H.Y.); yskim527@kiom.re.kr (Y.S.K.); hjyang@kiom.re.kr (H.J.Y.); wkcho@kiom.re.kr (W.-K.C.)

**Keywords:** chondrocyte, mast cells, mast cell-derived inflammatory mediator, matrix metalloprotease, *Viscum coloratum* extract

## Abstract

The accumulation and infiltration of mast cells are found in osteoarthritic lesions in humans and rodents. Nonetheless, the roles of mast cells in osteoarthritis are almost unknown. Although *Viscum coloratum* has various beneficial actions, its effect on allergic and osteoarthritic responses is unknown. In this study, we established an in vitro model of mast cell-mediated osteoarthritis and investigated the effect of the ethanol extract of *Viscum coloratum* (VEE) on IgE/antigen (IgE/Ag)-activated mast cells and mast cell-derived inflammatory mediator (MDIM)-stimulated chondrocytes. The anti-allergic effect of VEE was evaluated by degranulation, inflammatory mediators, and the FcεRI signaling cascade in IgE/Ag-activated RBL-2H3 cells. The anti-osteoarthritic action of VEE was evaluated by cell migration, and the expression, secretion, and activity of MMPs in MDIM-stimulated SW1353 cells. VEE significantly inhibited degranulation (IC_50_: 93.04 μg/mL), the production of IL-4 (IC_50_: 73.28 μg/mL), TNF-α (IC_50_: 50.59 μg/mL), PGD_2_ and LTC_4_, and activation of the FcεRI signaling cascade in IgE/Ag-activated RBL-2H3 cells. Moreover, VEE not only reduced cell migration but also inhibited the expression, secretion, and/or activity of MMP-1, MMP-3, or MMP-13 in MDIM-stimulated SW1353 cells. In conclusion, VEE possesses both anti-allergic and anti-osteoarthritic properties. Therefore, VEE could possibly be considered a new herbal drug for anti-allergic and anti-osteoarthritic therapy. Moreover, the in vitro model may be useful for the development of anti-osteoarthritic drugs.

## 1. Introduction

Mast cells are associated with allergic diseases including asthma, allergic rhinitis, and autoimmune diseases [1]. Anaphylactic shock is a type I allergic reaction and [2] is closely related to the degranulation of immunoglobulin E/antigen complex (IgE/Ag)-activated mast cells expressing FcεRI receptors as the high-affinity IgE receptors [3]. Mast cells contain numerous granules including histamine, inflammatory cytokines, prostaglandins, and leukotrienes [3]. Moreover, they are associated with initiation and/or aggravation of autoimmune diseases such as rheumatoid arthritis [1], which is included in osteoarthritis and the type III allergic class of conditions [2]. Accumulation and infiltration of mast cells are found in joint tissues and fluids in humans [4] and rodents [5,6] with rheumatoid arthritis; however, the roles of mast cells in osteoarthritis are almost unknown.

Mistletoe is a semi-parasitic plant that possesses various beneficial effects such as anticancer, anti-inflammation, anti-obesity, antioxidant, and neuroprotection [7]. *Viscum coloratum*, known as Korean mistletoe, and *Loranthus parasiticus*, known as Mulberry mistletoe, have long been used as part of traditional medicine in Northeast Asia [8]. Extract of *Viscum album* L., known as European mistletoe, has been used as an anticancer therapy in Europe because lectins, carbohydrate-binding proteins, viscotoxins, and small phytoproteins in *Viscum album* L. possess strong anticancer activity [9]. Therefore, the beneficial effects of mistletoe are related to bioactive compounds including phenolic compounds and flavonoids [9,10,11,12,13]. Actually, phenolic compounds and flavonoids are known to be associated with anti-allergic activity in IgE/Ag-activated mast cells [14,15,16]. Therefore, ethanol extract of *Viscum coloratum* (VEE) may have anti-osteoarthritic properties and can potentially be used as a new herbal medicine for anti-osteoarthritic therapy.

In the present study, we observed that VEE exerted more potent anti-allergic activity than aqueous extract of *Viscum coloratum* (VAE) in IgE/Ag-activated mast cells. Thus, we hypothesized that VEE could inhibit activity and/or expression of matrix metalloproteases (MMPs), which induce cartilage degradation [17] in mast cell-derived inflammatory mediator (MDIM)-stimulated chondrocytes. To investigate the anti-allergic activity of VEE in IgE/Ag-activated mast cells, degranulation was measured by the activity of β-hexosaminidase, and the amounts of proinflammatory mediators such as tumor necrosis factor-α (TNF-α), interleukin-4 (IL-4), prostaglandin D_2_ (PGD_2_), and leukotriene C_4_ (LTC_4_) were measured using enzyme-linked immunosorbent assay (ELISA) and enzyme immunoassay (EIA) kits. To examine the anti-osteoarthritic action of VEE in MDIM-activated chondrocytes, cell migration, which is associated with the activation of MMPs [18], was evaluated by a wound healing assay and a Transwell migration assay. The activity of MMPs was evaluated by zymography. Finally, to elucidate the anti-allergic mechanisms and anti-osteoarthritic action of VEE, the above mechanism-related proteins and anti-allergic phytochemicals were analyzed using immunoblot analysis and ultra-high-performance liquid chromatography-quadrupole-exactive orbitrap-mass spectrometry (UHPLC-Q-Exactive Orbitrap-MS) analysis, respectively.

Herein, we established an in vitro model of mast cell-mediated osteoarthritis using both RBL-2H3 cells and SW1353 cells. The results showed that VEE could block MDIM-mediated osteoarthritis in chondrocytes by regulating the expression, secretion, and/or activities of MMP-1, MMP-3, and MMP-13. Such effects of VEE may provide further information for the development of a phytomedicine for anti-allergic and anti-osteoarthritic therapies. Moreover, the in vitro model may be useful in the development of a drug candidate that can exert anti-osteoarthritic properties.

## 2. Results

### 2.1. Inhibitory Effects of VAE or VEE on the IgE/Ag-Mediated Allergic Response in RBL-2H3 Cells

**Figure 1:**
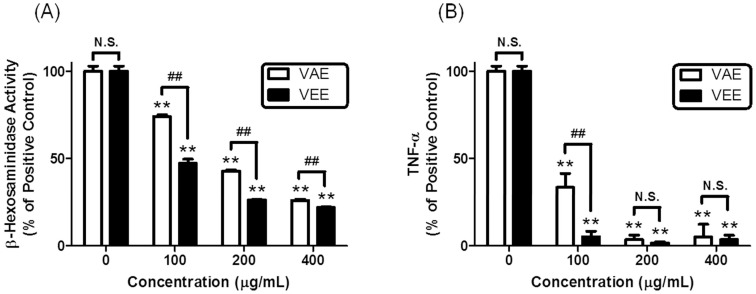
Inhibitory effects of VAE and VEE on degranulation and TNF-α formation in IgE/Ag-activated RBL-2H3 cells. RBL-2H3 cells were seeded in a 24-well plate (1 × 10^5^ cells/well) in MEM-α with 10% FBS at 37 °C overnight and further incubated with DNP-IgE for 24 h. IgE-sensitized cells were preincubated with VAE or VEE (0–400 μg/mL) for 1 h and then stimulated with DNP-HSA (0.1 μg/mL) for 4 h. β-Hexosaminidase activity and TNF-α levels were determined as described in Materials and Methods. The data are expressed as the mean ± SD values of triple determinations. N.S., not statistically significant; ** *p <* 0.01 versus 0 μg/mL group; ## *p <* 0.01 versus each concentration group. (**A**) β-Hexosaminidase; (**B**) TNF-α.

First, to investigate the effects of VAE and VEE on the IgE/Ag-mediated allergic response in mast cells, IgE-sensitized RBL-2H3 cells were incubated with various concentrations of VAE or VEE before antigen challenge (0.1 μg/mL dinitrophenyl-human serum albumin (DNP-HSA)). VEE showed stronger inhibitory actions in β-hexosaminidase activity and TNF-α formation than VAE (Figure 1). These results indicated that VEE includes richer anti-allergic phytochemicals than VAE. Therefore, the ethanol extract method is useful for developing a functional food or a plant-derived drug of *Viscum coloratum*.

### 2.2. Inhibitory Effects of VEE on the Formation of Inflammatory Mediators

**Figure 2:**
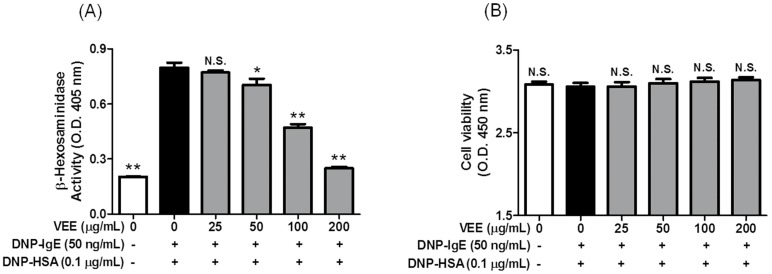

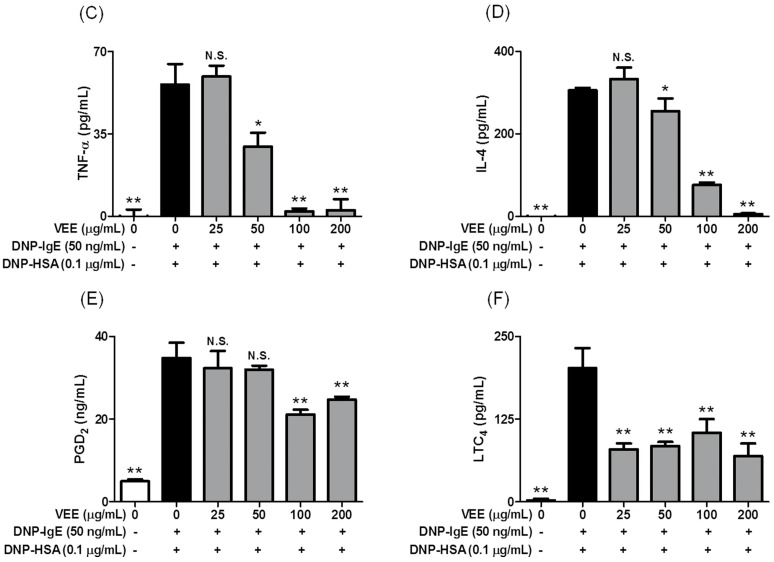
Effects of VEE on degranulation, inflammatory mediators and cell viability in IgE/Ag-activated RBL-2H3 cells. IgE-sensitized RBL-2H3 cells were preincubated with VEE (0–200 μg/mL) for 1 h prior to antigenic challenge. β-Hexosaminidase activity, the amounts of IL-4, TNF-α, PGD_2_, and LTC_4_, and cell viability were determined as described in the Materials and Methods section. The data are expressed as the mean ± SD values of triple or octuplex determinations. N.S., not statistically significant; * *p <* 0.05 and ** *p <* 0.01 versus DNP-HSA-treated group. (**A**) β-Hexosaminidase; (**B**) cell viability; (**C**) TNF-α; (**D**) IL-4; (**E**) PGD_2_; (**F**) LTC_4_.

Because we found stronger anti-allergic actions of VEE than VAE, we investigated the effects of VEE on the formation of inflammatory mediators and cell viability. Inflammatory mediators such as inflammatory cytokines, prostaglandins, and leukotrienes are produced and released from IgE/Ag-activated mast cells [3,19,20]. Moreover, they are related to the initiation and/or progression of allergic diseases such as asthma, allergic rhinitis, and rheumatoid arthritis [4,5,6,19,20]. As shown in Figure 2, VEE inhibited the formation of TNF-α (IC_50_ value, 50.59 μg/mL), IL-4 (IC_50_ value, 73.28 μg/mL), PGD_2_ and LTC_4_ in a dose-dependent manner. Additionally, VEE inhibited degranulation (IC_50_ value, 93.04 μg/mL) within non-cytotoxic concentrations in IgE/Ag-activated RBL-2H3 cells. Notably, VEE at 25 μg/mL exerted a strong inhibitory effect on LTC_4_ biosynthesis, although the inhibitory effect of VEE was not improved in concentrations exceeding 25 μg/mL. In contrast, VEE significantly inhibited PGD_2_ production in concentrations in excess of 100 μg/mL, although it weakly inhibited the formation of PGD_2_ in comparison to LTC_4_. Taken together, these results suggest that VEE can suppress allergic inflammation by inhibiting degranulation and formation of inflammatory mediators in IgE/Ag-activated mast cells. Therefore, VEE may regulate the initiation and progression of allergic diseases related to mast cells.

### 2.3. Effects of VEE on the Activation of FcεRI and Arachidonate Cascades

**Figure 3:**
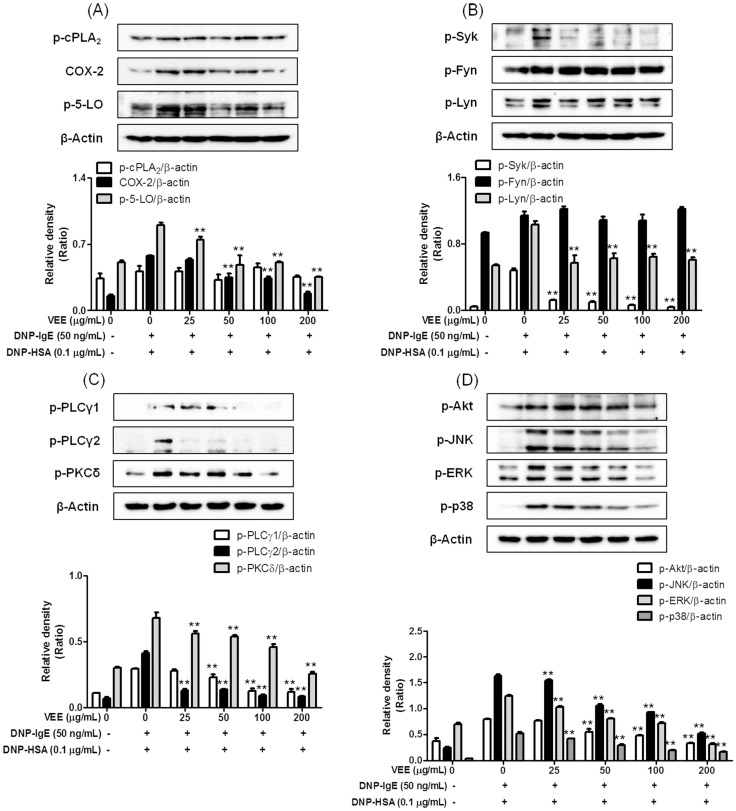
Effects of VEE on activation of the FcεRI and arachidonate cascades. RBL-2H3 cells were seeded in a 6-well plate (5 × 10^5^ cells/well) in MEM-α with 10% FBS at 37 °C overnight and further incubated with DNP-IgE for 24 h. IgE-sensitized RBL-2H3 cells were preincubated with VEE for 1 h and then stimulated with antigen for 4 h or 10 min. The cells were washed with 1× DPBS and lysed with a cell lysis buffer. The expression of p-cPLA_2_, COX-2, p-5-LO, p-Syk, p-Fyn, p-Lyn, p-PLCγ1, p-PLCγ2, p-PKCδ, p-Akt, p-JNK, p-ERK, p-p38, or β-actin was determined as described in the Materials and Methods section. Similar results were obtained in three independent experiments. ** *p <* 0.01 versus DNP-HSA-treated group. (**A**) p-cPLA_2_, p-5-LO, and COX-2; (**B**) p-Syk, p-Fyn, and p-Lyn; (**C**) p-PLCγ1/2 and p-PKCδ; (**D**) p-Akt, p-JNK, p-ERK, and p-p38.

Next, we further examined the effects of VEE on FcεRI and arachidonate cascades since VEE decreased the production of IL-4, TNF-α, PGD_2_, and LTC_4_ in IgE/Ag-activated mast cells. The biosynthesis of inflammatory mediators is associated with activation of FcεRI and arachidonate cascades [3]. Actually, both formation of inflammatory mediators and activation of FcεRI and arachidonate cascades are reduced by drug candidates in IgE/Ag-activated mast cells [14,15,16,21,22]. In the arachidonate cascade (DNP-HSA stimulation for 4 h), VEE significantly blocked the expression of COX-2 and the phosphorylation of 5-lipoxygenase (5-LO); however, it did not block the phosphorylation of cPLA_2_ (Figure 3A). In the FcεRI cascade (DNP-HSA stimulation for 10 min), VEE not only dramatically inhibited the phosphorylation of spleen tyrosine kinase (Syk) (Figure 3B) and PLCγ2 (Figure 3C), but it also dose-dependently reduced the phosphorylation of PLCγ1, PKCδ (Figure 3C), Akt, JNK, ERK, and p38 (Figure 3D) and partially suppressed the phosphorylation of Lyn, but not Fyn (Figure 3A). These findings suggest that VEE regulates the activation of arachidonate and FcεRI cascades by inhibiting the activation of Syk, COX-2, and 5-LO in IgE/Ag-activated mast cells. Although VEE dose-dependently suppresses both COX-2 expression and 5-LO phosphorylation, it cannot completely inhibit the formation of PGD_2_ and LTC_4_ at 200 μg/mL. It is suggested that VEE cannot affect the activity or expression of PGD and LTC_4_ synthase.

### 2.4. Effects of VEE on Cell Migration, and the Expression, Secretion, and/or Activity of MMPs in MDIM-Activated SW1353 Cells

**Figure 4:**
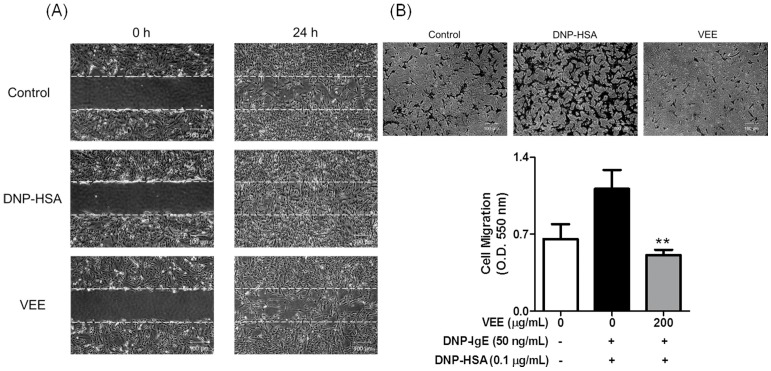
Inhibitory effect of VEE on cell migration of MDIM-stimulated SW1353 cells. SW1353 cells were seeded in a 60-mm dish (4 × 10^4^ cells/dish) and then incubated until 95% confluence or seeding occurred on a 24-well Transwell insert (1 × 10^5^ cells/well) in serum-free DMEM. The cells were then incubated with cultured media of RBL-2H3 cells for 24 h. A wound healing assay and a Transwell migration assay were performed as described in the Materials and Methods section. The data are expressed as the mean ± SD values of quadruple determinations. ** *p <* 0.01 versus DNP-HSA-treated group. (**A**) Wound healing assay; (**B**) Transwell migration assay.

**Figure 5:**
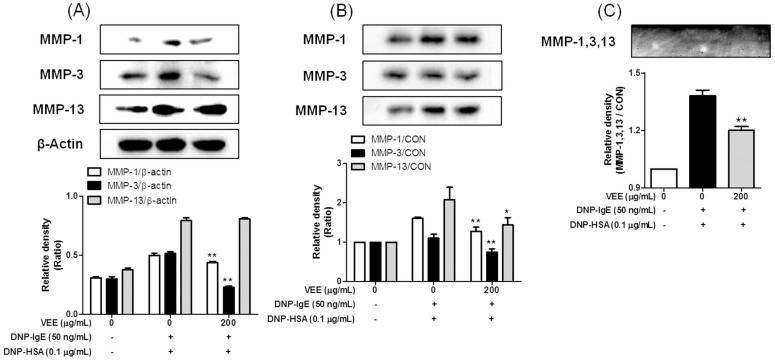
Effects of VEE on the expression, secretion, and activity of MMP-1, MMP-3, or MMP-13 in MDIM-activated SW1353 cells. SW1353 cells were seeded on a 60-mm dish and then incubated until 95% confluence. Precipitation of secreted proteins, a zymography assay, and immunoblotting analysis were determined as described in the Materials and Methods section. Similar results were obtained in three independent experiments. * *p <* 0.05 and ** *p <* 0.01 versus DNP-HSA-treated group. (**A**) Cell lysate; (**B**) cultured media; (**C**) zymogram.

Furthermore, we were interested in whether VEE affected the activity and/or expression of MMPs in MDIM-activated chondrocytes because mast cells are related to the initiation and aggravation of osteoarthritis including rheumatoid arthritis [1,4,5,6]. The activity and expression of MMPs are elevated in the cartilage tissues and joint fluids of humans [4] and rodents [5,6] with rheumatoid arthritis. Furthermore, MMPs induce cell migration [18] and cartilage degradation [17]. Thus, we investigate whether VEE could inhibit cell migration, and the expression, secretion, and/or activity of MMP-1, MMP-3, and MMP-13 in MDIM-activated SW1353 cells. VEE not only inhibited cell migration (Figure 4) but also reduced secretion and activity of MMP-1, MMP-3, or MMP-13 (Figure 5B,C). Additionally, VEE suppressed the expression of MMP-1 and MMP-3, but did not affect the expression of MMP-13 (Figure 5A). These results suggest that VEE exerts anti-osteoarthritic actions by regulating the expression, secretion, and/or activity of MMPs in MDIM-activated chondrocytes. Therefore, such effects of VEE may attenuate initiation and/or progression of MDIM-mediated osteoarthritis in osteoarthritic diseases.

### 2.5. Chemical Profile of the Active Compounds in VEE

**Figure 6:**
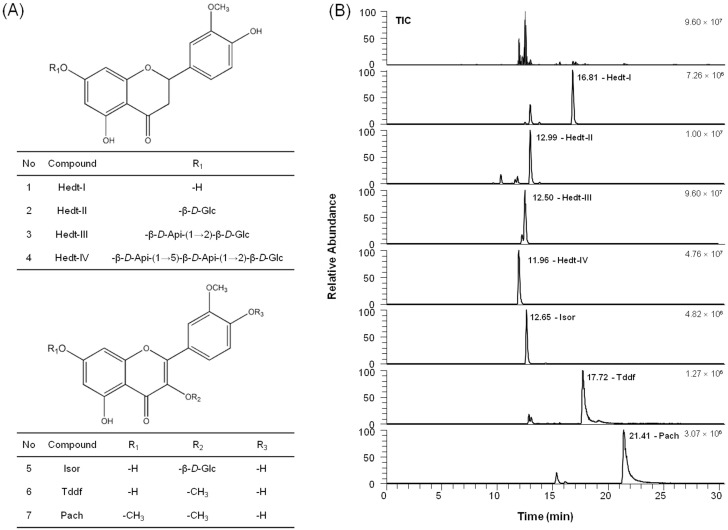
The chemical structures and the PRM chromatograms in the negative mode of VEE. The structures and PRM chromatograms of seven compounds in VEE are shown. Hedt-I, Hedt-II, Hedt-III, Hedt-IV, Isor, Tddf, and Pach were identified. UHPLC-Q-Exactive Orbitrap-MS analysis was performed as described in the Materials and Methods section. (**A**) The chemical structures; (**B**) PRM chromatograms.

Finally, to substantiate what underlies the anti-allergic and anti-osteoarthritic actions of VEE, we determined the total amounts of phenolic compounds and flavonoids in VEE. The reason is that *Viscum coloratum* includes various bioactive compounds such as lectins, viscotoxins, alkaloids, terpenes, sesquiterpene lactones, flavonoids, and phenolic compounds [9,10,11,12,13]. VEE included the following: total phenolic compounds (11.14 ± 0.05 mg/g dry weight, the mean ± SD values of triple determinations) and flavonoids (83.51 ± 0.56 mg/g dry weight, the mean ± SD values of triple determinations). Furthermore, we identified the flavonoid composition in VAE and VEE using the UHPLC-Q-Exactive Orbitrap-MS system. As shown in Figure 6B, the retention times for the peaks of homoeriodictyol (Hedt-I); homoeriodictyol-7-*O*-β-d-glycoside (Hedt-II); homoeriodictyol-7-*O*-β-d-apiose (1→2)-β-d-glycoside (Hedt-III); homoeriodictyol-7-*O*-β-d-apiose (1→5)-β-d-apiose (1→2)-β-d-glycoside (Hedt-IV); isorhamnetin-3-*O*-β-d-glucoside (Isor); 5,7,4′-trihydroxy-3,3′-dimethoxyflavone (Tddf); and pachypodol (Pach) were 16.81, 12.99, 12.50, 11.96, 12.65, 17.72, and 21.41 min, respectively. The peaks of Hedt-I, Tddf, and Pach, which are the aglycones in VEE, were higher than VAE, whereas the peaks of Hedt-II, Hedt-III, Hedt-IV, and Isor, which are the glycosides in VEE, were similar to VAE. Table 1 summarizes the LC-MS/MS data and the area ratio of the peaks in VEE. These results indicate that VEE includes richer aglycones than glycosides. Therefore, the aglycones may be responsible for the anti-allergic and anti-arthritic properties.

## 3. Discussion

*Viscum coloratum* and *Loranthus parasiticus* have been generally used as part of traditional medicine in Northeast Asia for centuries. These compounds possess multiple beneficial properties such as anticancer, anti-inflammation, anti-obesity, antioxidant, and neuroprotection [7]. Such effects of *Viscum coloratum* are associated with some bioactive compounds including flavonoids, lectins, phenolic compounds, sesquiterpene lactones, terpenes, and viscotoxins [9,10,11,12,13]. Recently, we showed that the aqueous extract of *Loranthus parasiticus* had anti-allergic actions in the IgE/Ag-mediated allergic responses in mast cells [23]. Moreover, activation of IgE-sensitized mast cells in articular tissues is associated with the initiation and/or aggravation of osteoarthritic diseases including rheumatoid arthritis [1]. Nonetheless, the effect of *Viscum coloratum* on the allergic response and osteoarthritis is still unknown.

Mast cells are closely associated with various allergic diseases such as asthma, allergic rhinitis and autoimmune diseases [1] and possess FcεRI receptors located on the extracellular surface [3]. IgE/Ag-activated mast cells release numerous inflammatory mediators such as histamine, inflammatory cytokines, prostaglandins, and leukotrienes, which participate in the acute or chronic inflammatory responses in surrounding normal tissues [3]. Moreover, accumulation and infiltration of mast cells are found in cartilage tissues and synovial fluids in humans [4] and rodents [5,6] with rheumatoid arthritis belonging to the type III allergic class [2]. The roles of recombinant IL-1β and TNF-α in osteoarthritis are well elucidated in in vitro and in vivo studies [24]. Additionally, their receptors are expressed on the surface of cartilage tissues in osteoarthritis [24]. Moreover, IL-1β inhibits the expression of multiple genes associated with the differentiation of chondrocytes [24]. Both IL-1β and TNF-α not only suppress the biosynthesis of type II collagen [24] but also upregulate the expression of MMPs in chondrocytes [25]. Subsequently, elevated MMPs in cartilage tissues and synovial fluids of patients with osteoarthritis reduce cartilage tissues by degrading collagen fibers [25]. Moreover, IL-1β and TNF-α induce activation of synovial fibroblasts and osteoclast cells that are associated with chronic inflammation and bone loss in osteoarthritis [26]. Collectively, we hypothesized that VEE can exert anti-osteoarthritic properties in MDIM-activated chondrocytes by regulating activation of IgE/Ag-stimulated mast cells. In this regard, VEE not only showed anti-allergic actions in IgE/Ag-activated RBL-2H3 cells but also suppressed anti-osteoarthritic properties in MDIM-activated SW1353 cells. Therefore, regulating IgE/Ag-activated mast cells is a key focus for prevention or treatment of osteoarthritis. In addition, such effects of VEE may be caused by phenolic compounds and flavonoids such as Hedt-I, Tddf, or Pach, which have antioxidant, anti-inflammatory, and antiviral activities [27,28,29].

Concerning the mechanisms responsible for the anti-allergic actions of VEE, one possible mechanism is associated with a direct inhibition of the FcεRI signaling cascade [3]. Degranulation and formation of inflammatory mediators in IgE/Ag-activated mast cells are correlated with the activation of the FcεRI receptors because clustering of FcεRI receptors by antigens initiates both the degranulation process and the biosynthesis of inflammatory mediators by activation of Syk, a rate-limiting protein in the FcεRI signaling cascade [3]. Therefore, Syk is an important signaling mediator in the early stage of the FcεRI signaling cascade. VEE was found to not only inhibit the phosphorylation of Syk, but also reduce the phosphorylation of PLCγ1/2 and PKCδ, which are associated with the degranulation process [3]. Additionally, VEE reduced the phosphorylation of Akt, ERK, JNK, and p38, which are associated with the production of inflammatory cytokines such as IL-4, TNF-α, and IL-1β [3]. Therefore, Syk is a target for the anti-allergic properties of VEE.

Another possible mechanism for the anti-allergic actions of VEE may be associated with reducing the activation of the arachidonate cascade in IgE/Ag-activated mast cells, which release and produce inflammatory lipid mediators such as PGD_2_, PGE_2_, LTB_4_, and LTC_4_ [30]. Therefore, the regulation of the arachidonate cascade is another important function related to the anti-allergic properties of VEE. In support of this information, VEE decreased both the release and production of PGD_2_ and LTC_4_, which are implicated in acute or chronic inflammation in allergic diseases such as asthma and allergic rhinitis [30]. In addition, VEE reduced both the expression of COX-2 as a rate-limiting enzyme of prostaglandin biosynthesis [30] and the phosphorylation of 5-LO as a rate-determining enzyme of leukotriene biosynthesis [30]. Thus, COX-2 and 5-LO are additional targets for the anti-allergic properties of VEE.

## 4. Materials and Methods

### 4.1. Reagents

DMEM, MEM-α medium, 1 × DPBS, antibiotics, and fetal bovine serum (FBS) were purchased from GE Healthcare Life Sciences (Hyclone™, Logan, UT, USA). The EZ-Cytox cell viability assay kit was obtained from Daeil Lab Service Co., Ltd. (Seoul, Korea). Specific antibodies against phospho-Akt, phospho-cPLA_2_, phospho-ERK, phospho-JNK, phospho-Lyn, phospho-p38, phospho-PKCδ, phospho-PLCγ1/2, phospho-Syk, and COX-2 were purchased from Cell Signaling Technology, Inc. (Beverly, MA, USA). A specific antibody against phospho-Fyn was obtained from Biorbyt Ltd. (Cambridge, UK). A specific antibody against β-actin was purchased from Santa Cruz Biotechnology, Inc. (Dallas, TX, USA). A specific antibody against phospho-5-LO and EIA kits for PGD_2_ and LTC_4_ were obtained from Cayman Chemical Co. (Ann Arbor, MI, USA). ELISA kits for IL-4 and TNF-α were purchased from e-Bioscience, Inc. (Science Center Drive, San Diego, CA, USA). A specific antibody against MMP-1 was obtained from R&D Systems, Inc. (Minneapolis, MN, USA). Specific antibodies against MMP-3 and MMP-13 were purchased from Abcam, Inc. (Cambridge, UK). 4-Nitrophenyl *N*-acetyl-β-d-glucosaminide (p-NAG), casein, DNP-HSA, DNP-IgE, trichloroacetic acid, caffeic acid, diethylene glycol, Folin-Ciocalteu reagent, and quercetin were obtained from Sigma-Aldrich Co. (St. Louis, MO, USA). All other chemicals were of analytical grade.

### 4.2. Preparation of Viscum Coloratum Extracts

VAE and VEE were prepared according to a modification of a process previously reported [16]. *Viscum coloratum* (1 kg) was boiled in distilled water or 70% ethanol (10 L) for approximately 3 h at 115 °C. The aqueous extract was filtered through a testing sieve (Aperture 500 μm and 150 μm) and a nylon net filter (Aperture 60 μm; Millipore Co., Denver, MA, USA) and then deposited overnight. The supernatant was lyophilized, and then the dried pellet was stored at −20 °C until use. VAE and VEE were dissolved in 10% dimethyl sulfoxide (DMSO) solution for all experiments.

### 4.3. Determination of Total Phenolic and Flavonoid Compounds

The total amounts of phenolic compounds and flavonoids in VEE were evaluated following a previously reported method [14]. To measure the total amount of phenolic compounds, 0.33 mL of the sample solution (100 mg/mL) was mixed with 2.5 mL of distilled water and then incubated with 0.16 mL of Folin-Ciocalteu reagent for 5 min. The above solution was further incubated for 30 min in darkness after treatment with 10% sodium bicarbonate solution (0.3 mL). The absorbance at 760 nm was measured using a microplate reader (SpectraMax i3, Molecular Devices, Sunnyvale, CA, USA). A standard curve was prepared to express the results as caffeic acid equivalents. To determine the total amount of flavonoids in VEE, 0.4 mL of VEE was added to 4 mL of 90% diethylene glycol containing 0.1 N NaOH, and then the mixture was incubated for 1 h. The absorbance of the solution at 420 nm was measured using a microplate reader. A standard curve was prepared to express the results as quercetin equivalents.

### 4.4. Analytical Methods

To analyze active compounds in VAE and VEE, the UHPLC analysis was performed using a Dionex Ultimate 3000 system equipped with a quaternary pump, an autosampler, and a column compartment (Thermo Fisher Scientific Inc., Sunnyvale, CA, USA) and a Kinetex^®^ 2.6 µm C18 100 Å column (100 × 4.6 mm I.D., 2.6 μm, Phenomenex, Torrance, CA, USA). The active phytochemicals were eluted in a gradient system composed of solvent A (0.1% formic acid) and solvent B (acetonitrile). The gradient was 10-10-80-80-10-10% of solvent B at gradient time, *t*G = 0-2-22-25-25.5-30.5 min, respectively. The column oven temperature was 40 °C, and the flow rate was 0.3 mL/min. An injection volume of 5 μL was applied. The powder of VAE or VEE was weighed exactly and dissolved in methanol at a concentration of 10 mg/mL, centrifuged (8000× *g*) for 10 min, and then the supernatant was filtered through a 0.2-μm membrane filter (Whatman International Ltd., Maidstone, UK) prior to injection. All sample solutions were stored at 4 °C before use.

To identify the active compounds in the solutions, liquid chromatography-tandem mass spectrometry (LC-MS/MS) analysis was performed with a Q-Exactive Orbitrap-MS (Thermo Fisher Scientific Inc., Waltham, MA, USA) coupled with an electrospray ionization source for the ionization of the target components in the negative ion mode. MS/MS data processing was carried out using Xcalibur 3.0 software (Thermo Fisher Scientific Inc., Waltham, MA, USA). High-resolution full MS and parallel reaction monitoring (PRM) scan modes were applied to identify the target components. The operated parameters were as follows: spray voltage, 2.80 kV; sheath gas pressure, 40 psi; auxiliary gas pressure, 5 arb; capillary temperature, 320 °C; S-lens RF level, 60.0; auxiliary gas heater temperature, 300 °C; scan modes, full MS (resolution, 70,000) and MS/MS (resolution, 17,500; AGC target value, 1e6; normalized collision energy and stepped normalized collision energy, 30 eV) and scan range, *m*/*z* 150–1000. MS spectra were acquired on the centroid mode.

### 4.5. Cell Culture

RBL-2H3 cells, a mast cell line originating from rat basophilic leukemia [31], or SW1353 cells, a chondrocyte cell line originating from human chondrosarcoma [32], were obtained from the Korean Cell Line Bank (Seoul, Korea) or the American Type Culture Collection (Manassas, VA, USA). The cells were cultured in MEM-α or DMEM, respectively, which contained 10% (*v*/*v*) FBS with antibiotics, at 37 °C in a humidified atmosphere of 5% CO_2_. All the experiments used a vehicle control group containing 0.1% DMSO.

### 4.6. Cell Viability Assay

Cell viability was determined following a previous method [16]. IgE-sensitized RBL-2H3 cells were preincubated with VEE (0–200 μg/mL) in MEM-α containing 1% FBS for 1 h. The cells were then simultaneously mixed with 0.1 μg/mL DNP-HSA and 10 μL EZ-Cytox reagent and further incubated for 4 h. Cell viability was determined at 450 nm using a microplate reader.

### 4.7. β-Hexosaminidase Activity Assay

β-Hexosaminidase activity was evaluated following the previously reported method [15]. Supernatant (25 μL) was mixed with 50 μL p-NAG (10 mM) in 0.1 M sodium citrate buffer (pH 4.5) and then incubated for 1 h at 37 °C. The reaction was terminated by 0.1 M sodium carbonate buffer (pH 10.0). The absorbance was measured at 405 nm using a microplate reader.

### 4.8. Enzyme-Linked Immunosorbent Assay for TNF-α and IL-4

To determine the amounts of TNF-α or IL-4 in cultured media, all the cultured media were centrifuged (17,000× *g* at 4 °C) for 10 min and then stored at −80 °C until use. IL-4 and TNF-α were detected by ELISA kits according to the manufacturer’s instructions.

### 4.9. Enzyme Immunoassay Analysis for PGD_2_ and LTC_4_

To measure the levels of PGD_2_ or LTC_4_ in cultured media, all cultured media were centrifuged and stored at −80 °C until use. PGD_2_ and LTC_4_ were measured by EIA kits according to the manufacturer’s instructions.

### 4.10. Cell Migration

IgE-sensitized RBL-2H3 cells were preincubated with or without 200 μg/mL VEE in serum-free DMEM for 1 h prior to antigen challenge and then incubated for an additional 4 h. All cultured media were centrifuged, and then the supernatant was transferred into 60-mm dishes or Transwell plates. To evaluate cell migration using a wound healing assay, SW1353 cells were seeded on a 60-mm dish (4 × 10^4^ cells/dish), and then incubated until 95% confluence. An injury line (a width of 0.2 mm) on the cell monolayer was made with a sterile scraper, washed with 1× DPBS, and then incubated with the above cultured media for 24 h. Cell morphology was observed under light microscopy (40× magnification).

To confirm cell migration using a Transwell migration assay, the above cultured media (600 µL) were transferred into lower chambers of a 24-well Transwell insert plate (8.0 µm pore size, SPL Life science Co., Pocheon, Korea), and then SW1353 cells were seeded onto the 24-well Transwell inserts (1 × 10^5^ cells/well) in 100 µL serum-free DMEM at 37 °C. After 24 h, the insert insides were swabbed and then stained with 0.2% crystal violet in a 20% methanol solution. The stained cells were observed under light microscopy (40× magnification) and then incubated with 200 µL acetic acid. The absorbance was measured at 620 nm using a microplate reader.

### 4.11. Precipitation of Secreted Proteins

Secreted proteins in cultured media were concentrated according to a previously described method [33]. SW1353 cells were incubated with the cultured media of IgE/Ag-activated RBL-2H3 cells for 24 h. To concentrate secreted proteins derived from MDIM-stimulated SW1353 cells, the supernatant was mixed with 10% ice-cold trichloroacetic acid and then centrifuged. The pellet of secreted proteins was washed with acetone twice, dissolved in RIPA buffer, and stored at −20 °C until use.

### 4.12. Zymography Assay

The zymography assay was performed following a previously described method [34]. Cultured media of SW1353 cells were electrophoresed using an 8% polyacrylamide gel containing 0.1% casein. The gel was washed with washing buffer (50 mM Tris-HCl containing 100 mM NaCl and 2.5% Triton X-100, pH 7.5) for 30 min and then incubated in reaction buffer (50 mM Tris-HCl including 150 mM NaCl, 10 mM CaCl_2_, 1 μM ZnCl_2_, and 0.02% sodium azide, pH 7.5) for 48 h at 37 °C. Subsequently, the gel was stained with 0.2% Coomassie brilliant blue R-250 staining solution (Bio-Rad, Hercules, CA, USA) and then destained (10% isopropanol with 10% acetic acid). The activity of MMPs was detected using an imaging system (ChemiDoc Touch Imaging System, Bio-Rad, Hercules, CA, USA). The density of each inverted band was measured using ImageJ software (version 1.49v for Windows; NIH, Bethesda, MD, USA).

### 4.13. Immunoblotting Analysis

Immunoblotting analysis was conducted following a previously described method [15]. PVDF membranes containing blotted proteins were visualized using a chemiluminescent reaction (ECL Plus kit, Bio-Rad, Hercules, CA, USA) with the imaging system. The phosphorylation or expression levels of target proteins were compared to the loading control proteins (β-actin) and their densities were measured using ImageJ software, and the results are expressed as a density ratio of each protein identified using a protein standard-size marker (BIOFACT, Daejeon, Korea).

### 4.14. Statistical Analysis

The experimental results are reported as the mean ± SD. One-way analysis of variance (ANOVA) was used for multiple comparisons (GraphPad Prism version 5.03 for Windows, San Diego, CA, USA). The Dunnett test was applied for significant variations between treated groups. Differences at the * *p* < 0.05 and ** *p* < 0.01 levels were considered statistically significant.

## 5. Conclusions

The present study demonstrates that VEE not only exerts anti-allergic actions in IgE/Ag-activated mast cells, but also possesses anti-osteoarthritic actions in MDIM-activated chondrocytes. Such effects of VEE may be caused by Hedt-I, Tddf, or Pach. These findings revealed a novel feature of VEE in IgE/Ag-mediated allergic reactions and MDIM-mediated osteoarthritis. The mechanisms for its anti-allergic and anti-osteoarthritic actions may include multiple targets such as Syk, PLCγ1/2, PKCδ, Akt, ERK, JNK, p38, 5-LO, and COX-2 in IgE/Ag-activated mast cells, and MMP-1, MMP-3, and MMP-13 in MDIM-activated chondrocytes. Such effects of VEE provide further information for developing a functional food or a phytomedicine for mast cell-related diseases including asthma and rheumatoid arthritis. Moreover, the established in vitro model of osteoarthritis may be useful for developing a new drug for anti-osteoarthritic therapies. Further animal studies are necessary to confirm the anti-allergic and/or anti-osteoarthritic properties of VEE in an in vivo system.

## Figures and Tables

**Table 1:** The composition of flavonoids in *Viscum coloratum*.

No.	Compounds	*t*_R_ (min)	Formula	[M − H]^−^ Calculated (*m*/*z*)	[M − H]^−^ Detected (*m*/*z*)	Delta (ppm)	MS/MS (*m*/*z*)	Area Ratio (VEE/VAE)	
								Ratio (%)	SD
1	Hedt-I	16.81	C_16_H_14_O_6_	301.07176	301.07108	−0.043	151.00218	235.71	16.09
2	Hedt-II	12.99	C_22_H_24_O_11_	463.12458	463.12326	0.159	301.07114, 151.00224	96.24	5.25
3	Hedt-III	12.50	C_27_H_32_O_15_	595.16684	595.16547	−0.781	301.07104, 151.00211	126.39	6.01
4	Hedt-IV	11.96	C_32_H_40_O_19_	727.20910	727.20770	−0.747	301.07101	128.20	9.00
5	Isor	12.65	C_22_H_22_O_12_	477.10385	477.10291	−0.442	357.06079, 314.04269, 271.02429	162.03	8.45
6	Tddf	17.72	C_17_H_14_O_7_	329.06668	329.06619	0.281	314.04269, 299.0191	671.04	56.65
7	Pach	21.41	C_18_H_16_O_7_	343.08233	343.08142	−0.051	328.05823, 313.03488	994.33	78.50

The data are expressed as the mean ± SD values of quintuple determinations.
